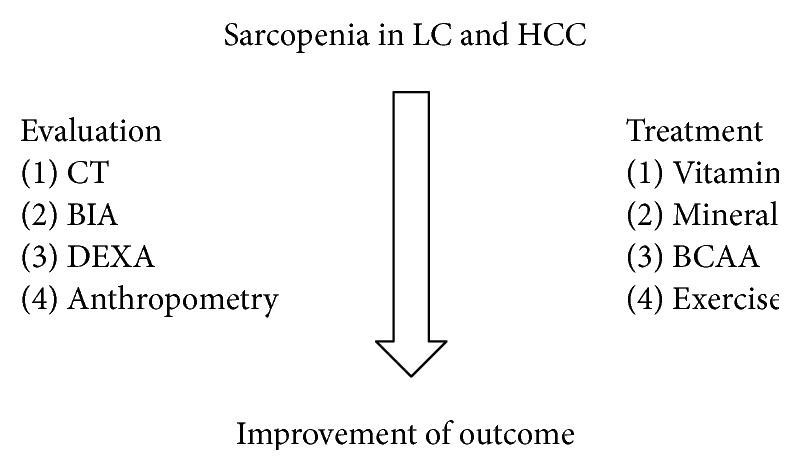# Liver Cirrhosis: Evaluation, Nutritional Status, and Prognosis

**DOI:** 10.1155/2015/872152

**Published:** 2015-09-30

**Authors:** Hiroki Nishikawa, Yukio Osaki

**Affiliations:** ^1^Department of Gastroenterology and Hepatology, Osaka Red Cross Hospital, Osaka, Japan; ^2^Division of Hepatobiliary and Pancreatic Disease, Department of Internal Medicine, Hyogo College of Medicine, Hyogo, Japan

## Abstract

The liver is the major organ for the metabolism of three major nutrients: protein, fat, and carbohydrate. Chronic hepatitis C virus infection is the major cause of chronic liver disease. Liver cirrhosis (LC) results from different mechanisms of liver injury that lead to necroinflammation and fibrosis. LC has been seen to be not a single disease entity but one that can be graded into distinct clinical stages related to clinical outcome. Several noninvasive methods have been developed for assessing liver fibrosis and these methods have been used for predicting prognosis in patients with LC. On the other hand, subjects with LC often have protein-energy malnutrition (PEM) and poor physical activity. These conditions often result in sarcopenia, which is the loss of skeletal muscle volume and increased muscle weakness. Recent studies have demonstrated that PEM and sarcopenia are predictive factors for poorer survival in patients with LC. Based on these backgrounds, several methods for evaluating nutritional status in patients with chronic liver disease have been developed and they have been preferably used in the clinical field practice. In this review, we will summarize the current knowledge in the field of LC from the viewpoints of diagnostic method, nutritional status, and clinical outcomes.

## 1. Introduction

The liver is the major organ for the metabolism of three major nutrients: protein, fat, and carbohydrate [1, 2]. Chronic hepatitis C virus (HCV) infection affects about 170 million people worldwide and is the most common cause of chronic liver disease. Of these HCV-infected individuals, 20–30% eventually develop liver cirrhosis (LC) or hepatocellular carcinoma (HCC). In our country, about 30,000 persons per year die from HCC, with 70–80% of these deaths ascribed to HCV [3, 4].

LC results from different mechanisms of liver injury that lead to necroinflammation and fibrosis. Histologically, LC is characterized by diffuse nodular regeneration surrounded by dense fibrotic septa with subsequent collapse of liver structures and thus causes pronounced distortion of vascular architecture in the liver [5].

Increasingly, LC has been seen to be not a single disease entity but one that can be graded into distinct clinical stages related to prognosis [5]. In addition, the economic and social burden of LC is immense considering decreased quality of life, the disability of labor, poorer physical activity, and need for frequent hospitalizations in patients with LC.

In terms of diagnostic methods for LC, several noninvasive methods have been developed and these methods have been used for predicting prognosis in patients with LC; these include serum markers such as aspartate aminotransferase to platelet ratio index (APRI), FIB-4 index, aspartate aminotransferase (AST) to alanine aminotransferase (ALT) ratio, or modalities such as acoustic radiation force impulse (ARFI), transient elastography (TE), and magnetic resonance elastography [6–13].

On the other hand, subjects with LC often have protein-energy malnutrition (PEM) and poor physical activity. These conditions often result in sarcopenia, which is the loss of skeletal muscle volume and increased muscle weakness. Recent studies have demonstrated that PEM and sarcopenia are predictive factors for poorer survival in patients with LC [14]. Based on these backgrounds, several methods for evaluating nutritional status in patients with chronic liver disease such as indirect calorimetry, dual-energy X-ray absorptiometry (DEXA), bioimpedance analysis (BIA), and anthropometry have been developed and they have been preferably used in the clinical settings [15].

In this review, we will summarize the current knowledge in the field of liver cirrhosis from the view of diagnostic method, nutritional status, and clinical outcomes.

## 2. Conventional Classification and Prognostic Assessment of Liver Cirrhosis

Currently, the most commonly used classification of liver function for patients with LC is Child-Pugh classification. This was originally designed to predict mortality during surgery in patients with LC [16]. This has been demonstrated to be useful in determining patient prognosis and thus several staging system for HCC including Japan Integrated Staging (JIS), Barcelona Clinic Liver Cancer (BCLC), and Cancer of Liver Italian program (CLIP) use this system as prognostic determinant [17–20]. The Model for End-Stage Liver Disease (MELD) score was originally developed as a prognostic model of early mortality in LC patients undergoing a transjugular intrahepatic portosystemic shunt (TIPS) [21]. This score includes variables of serum concentrations of bilirubin and creatinine and international normalized ratio for prothrombin time (INR) and, in most liver transplantation centers, MELD score has replaced the Child-Pugh score for priority of organ allocation due to superiority of prognostic ability of MELD score [21, 22]. On the other hand, serum sodium has been demonstrated to be an independent risk factor for mortality in LC patients with or without HCC [23, 24]. Kim et al. reported that the addition of the serum sodium to generate the MELD-Na score was more accurate than MELD for predicting short-term mortality on the waiting list of liver transplantation in LC patients [23]. Moreover, in their study, they estimated that the use of MELD-Na might have prevented 7% of deaths that occurred within 90 days of listing for liver transplantation [23]. In our previous study (*n* = 1170), we demonstrated that lower serum sodium concentration is a useful predictor in HCC patients complicating with LC [24]. Hepatic venous pressure gradient as determined by subtraction of the free-hepatic venous pressure from the wedged hepatic venous pressure has also been demonstrated to be an independent predictor in patients with LC [25]. However, unfortunately, these models did not include nutritional status of the LC patients.

On the other hand, D'Amico et al. have classified compensated LC into clinical stages 1 and 2 and decompensated LC in clinical stages 3 and 4 based on a systematic review of 118 reports [26]. They defined patients in clinical stage 1 as neither varices nor ascites and reported that the 1-year mortality was only 1%, and if patients develop varices (clinical stage 2), the 1-year mortality increases up to 3.4%. Furthermore, with decompensated LC and onset of ascites (clinical stage 3), the 1-year mortality increases up to 20%, and, following a variceal bleeding (clinical stage 4), the 1-year mortality was higher than 50% [26]. Currently, a proposal has been made to include two more additional clinical stages to this classification system: clinical stage 5, LC patients with bacterial infections (such as spontaneous bacterial peritonitis or bacteremia) as 1-year mortality increases from 49% to 66%, and clinical stage 6, patients with renal failure as mortality at 1-year could be around 70% [27, 28]. These classification systems are promising for predicting prognosis in patients with LC.

## 3. Noninvasive Methods for Predicting LC or LC Related Complications

Noninvasive markers of LC can be radiologic or serum based. Although liver biopsy remains the reference standard for evaluating the extent of liver fibrosis in patients with chronic liver diseases, several noninvasive methods such as TE and ARFI have been developed as alternatives to liver biopsies. Recent reports have focused on assessing the performance of noninvasive methods through long-term follow-up studies with clinical outcomes associated with LC [29–31].

Vergniol et al. reported that noninvasive tests for liver fibrosis (measurement of liver stiffness (FibroScan), FibroTest, APRI, and FIB-4 index) can predict 5-year survival of patients with chronic hepatitis C (*n* = 1457) [29]. Singh et al. demonstrated in their meta-analysis (*n* = 7058) that the degree of liver stiffness using elastography is associated with risk of decompensated cirrhosis, HCC, and death in patients with chronic liver diseases and it might be used in risk stratification [30]. A recent Japanese study demonstrated that measurements of spleen stiffness using ARFI can be used to identify patients with cirrhosis with esophageal varices (EVs) or high-risk EVs [31].

On the other hand, we previously reported that the GSA index as defined by the uptake ratio of the liver to the liver plus heart at 15 min to the uptake ratio of the heart at 15 min to that at 3 min ratio calculated from 99mTc-labeled diethylene triamine pentaacetate-galactosyl human serum albumin (99mTc-GSA) scintigraphy yielded the highest area under the receiver operating curve (AUROC) for predicting histologically proven cirrhosis with a level of 0.786 at an optimal cut-off value of 1.37 (sensitivity: 65.9%; specificity: 79.0%) in HCV-related HCC patient treated with surgical resection (SR) (*n* = 213) and it can be a useful predictor for HCC recurrence after surgery [32]. Furthermore, in non-B and non-C HCC patients treated with SR (*n* = 118), we have shown that the FIB-4 index yielded the highest AUROC for histologically proven cirrhosis with a level of 0.887 at an optimal cut-off value of 2.97 (sensitivity: 92.3%; specificity: 69.6%), and FIB-4 index >2.97 (*P* = 0.044) was a significant independent factor linked to HCC recurrence [33].

Recently, the Wisteria floribunda agglutinin-positive human Mac-2-binding protein (WFA+-M2BP) was demonstrated to be a liver fibrosis glycobiomarker with a unique fibrosis-related glycoalteration [34]. Yamasaki et al. reported that WFA+-M2BP can be applied as a useful surrogate marker for not only liver fibrosis but also the risk of HCC development [35].

## 4. Nutritional Status and Nutritional Assessment in Liver Cirrhosis

Cirrhosis, which develops over a long period of time, is frequently complicated with PEM [1, 2]. In our data, the proportion of PEM in LC patients was around 30% (unpublished data). PEM is one of the most common complications in LC patients and it is associated with high morbidity and mortality for patients with LC [1, 2, 36]. However, despite the significant role that the nutritional status has in the prognosis of LC, it is frequently overlooked as the nutritional assessment could be complex in LC patients with fluid retention and/or overweight [37].

Clinicians should consider various aspects of the LC patients including medical history, physical examination, the severity of underlying illness, and biochemical data for the nutritional evaluation [37]. The subjective global assessment (SGA) is one of commonly used instruments for nutritional assessment in patients with LC. In general, nutritional evaluation is performed using SGA, anthropometry including muscle arm circumference and body mass index (BMI), and biological markers such as albumin and prealbumin (transthyretin). The factors of the SGA consist of physical examination component that assesses the loss of subcutaneous fat, peripheral edema, and muscle wasting [38]. The quantity of muscle and subcutaneous tissue is graded subjectively by the examiner. Then, it is classified as normal, mildly, moderately, or severely decreased [38]. Considering multiple components such as body weight loss and clinical symptoms, the patients are graded as well nourished (SGA grade A), moderately malnourished or suspected of being malnourished (SGA grade B), or severely malnourished (SGA grade C) [38]. However, the SGA may be a partially subjective method. In addition, previous studies reported that SGA has shown low sensitivity in LC patients for nutritional diagnosis, as it underestimates the nutritional state in the majority of LC patients [39]. There is one interesting report of comparison between SGA grading system and a tool for controlling nutritional status (CONUT), which was proposed by Ignacio de Ulíbarri et al. [40, 41]. In that report, CONUT had the good agreement with SGA grading system (kappa index: 0.680) [41].

On the other hand, several laboratory tests have been used as a part of the nutritional assessment in patients with LC, including albumin, prealbumin, the prothrombin time, creatinine height index, and indirect evaluation of the of immune function [42–46]. However, as their studies had diverse results, an optimal index for nutritional status in LC patients in terms of availability, reproducibility, practicality, and prognostic performance is required [42–46].

Nutrition and exercise management can improve PEM and sarcopenia in patients with LC [14]. Nutritional management includes sufficient dietary intake and improved nutrient metabolism. However, with the current high prevalence of obesity, the number of obese LC patients has increased, and restriction of excessive caloric intake without the exacerbation of impaired nutrient metabolism is needed for LC patients with obesity [14]. Exercise management can increase skeletal muscle strength and volume.

## 5. Sarcopenia in Liver Cirrhosis

Sarcopenia is characterized by the depletion of skeletal muscle mass [47, 48]. In general, skeletal mass is maintained by a balance between synthesis and breakdown of protein [49]. LC patients have insufficient glycogen stores because of deterioration of liver function and energy generation pattern in these patients after an overnight fast is reported to be equivalent to that observed in healthy controls after 2 or 3 days of starvation [49]. These catabolic states increase the consumption of amino acids as an energy source and accelerate the breakdown in skeletal muscle to release amino acids, eventually leading to sarcopenia [49]. Recently, some studies have indicated that hyperammonemia can cause sarcopenia [50].

On the other hand, sarcopenia has become a key clinical entity for understanding the impact of aging on health outcomes. In 1989, Rosenberg first introduced the term “sarcopenia” to refer to age-related loss of skeletal muscle mass and volume [51]. Similar to bone, when persons reach around 50 years of age, they lose about 1-2% of their muscle mass per year [52]. Sarcopenia is a common disorder in aged populations contributing to functional decline, disability, and frailty [51, 52]. Several studies reported the increased risk of chronic metabolic disorders and mortality in persons with low muscle mass [53, 54]. Aging-related sarcopenia is defined as primary sarcopenia, whereas LC is a cause of secondary sarcopenia. Hiraoka et al. reported that in their analysed 988 subjects with chronic liver disease and 372 normal control subjects, presarcopenia as defined by less than two standard deviations below the mean psoas muscle area index (psoas muscle area at the mid-L3 level in CT (cm^2^)/height (m)^2^ value in the controls) was observed in 15.3% of patients with chronic hepatitis, 24.4% of those with Child-Pugh A, 37.7% of those with Child-Pugh B, and 37.1% of those with Child-Pugh C and the frequency of presarcopenia was higher in chronic hepatitis regardless of age as compared with normal controls [55].

### 5.1. Assessment Methods for Sarcopenia

Several methods for sarcopenia assessment in patients with LC have been proposed.

#### 5.1.1. Handgrip Strength

Hirsch et al. demonstrated in their controlled trial that handgrip strength was a useful marker for the assessment of nutritional status in LC patients [56]. Currently, The European Working Group on Sarcopenia in Older People (EWGSOP) recommends measurement of handgrip strength as a practical measure of muscle strength [57]. However, it should be kept in mind that this method has not been well established as considerable variation in the measurement methods has the potential to lead to measurement errors.

#### 5.1.2. Imaging Studies: CT

According to the recent investigations, the psoas muscle area or thickness can be measured on the axial CT scan at the various levels of lumbar spine such as L3 vertebral level, L4 vertebral level, and at the level of umbilicus for assessing sarcopenia [58–61]. These studies showed the good correlations of clinical outcomes and the psoas muscle mass [58–61]. The psoas muscle can be easily identified and easily measured on a CT scan, as it is surrounded by retroperitoneal fat tissue and vertebra, and is not susceptible to the compression of ascites or splenomegaly [58–61]. Thus, measurement of psoas muscle area at L3 vertebral level has been preferably used for assessing sarcopenia. However, no consensus value for CT-based sarcopenia has been well established in Asian populations.

#### 5.1.3. Bioimpedance Analysis

BIA is a noninvasive technique that measures electrical resistance and reactance [39, 62–64]. In recent studies, electrical BIA has been proposed for body composition analysis in patients with chronic liver disease [39, 62–64]. This method is based on the principle that body fat and no fat mass have specific components, such as water, proteins, and minerals [39, 62–64]. Electrical bioimpedance consists in the delivery of a low-intensity electric current which flows through the body by the ions movements [39, 62–64]. Fernandes et al. reported that the assessment using BIA presented a statistically significant correlation with Child-Pugh classification [39]. On the other hand, BIA did not demonstrate the ability to distinguish between minimal and advanced degrees of hepatic fibrosis in patients with chronic HCV infection [63].

#### 5.1.4. Dual-Energy X-Ray Absorptiometry

DEXA through a low-dose X-ray can be used to measure fat, total body bone mineral, and fat-free soft tissue mass [65, 66]. In healthy persons, excellent agreement is found between data obtained using DEXA and data obtained from the more established reference methods [66]. However, this technique is not accurate for evaluating body composition in LC patients with fluid retention.

#### 5.1.5. Other Methods

Skin-fold thickness measurement using a caliper is the method that quantify fat mass in the upper arm (midarm muscle area) [67]. However, there have been conflicting reports for the accuracy for predicting malnutrition in LC patients because of its interobserver variability, and this method did not correlate with Child-Pugh classification [66, 67].

## 6. Sarcopenic Obesity 

The current global obesity epidemic has created a new condition: the combination of obesity and sarcopenia, described as sarcopenic obesity [68]. As LC patients occasionally have sarcopenia (around 40%) and obesity (around 30%), it can be deduced that a considerable number of LC patients may have sarcopenic obesity [69]. In addition, obesity is often accompanied by nonalcoholic fatty liver disease (NAFLD), and the prevalence of this liver disease is increasing in industrialized countries. NAFLD can progress to nonalcoholic steatohepatitis and LC [70]. The increase in obesity prevalence rates in elderly patients is also of concern, given the associated disease risks such as coronary heart disease and more limited treatment options available in this age group. Sarcopenic obesity has been also found to be related to poorer survival in patients with solid tumors of the respiratory and gastrointestinal tracts [71]. Sarcopenic obesity may become a major condition in LC patients in the future.

Sarcopenic obesity is assuming a significant role as a risk factor due to the double metabolic burden derived from excess adiposity (obesity) and low muscle mass (sarcopenia). Obesity also induces systemic inflammation and insulin resistance and both prompt hypercatabolism and impairs the anabolic effect of muscles, leading to protein breakdown stimulation and muscle synthesis suppression [53]. Skeletal muscle plays a significant role in insulin sensitivity as a primary tissue associated with whole body insulin-mediated glucose uptake [53]. Several studies reported that low skeletal muscle mass is linked to obesity, metabolic syndrome, and dysglycemia, and the reverse was demonstrated in large populations with higher muscle mass associated with better insulin resistance and a lower risk of developing diabetes [53, 54, 72]. Moreover, a recent study demonstrated that sarcopenic obesity is more closely linked to insulin resistance than obesity or sarcopenia alone [54]. Taken together, this new condition may lead to accelerating sarcopenia progression.

On the other hand, sarcopenic obesity is a newly recognized clinical entity following living donor liver transplantation [73]. Choudhary et al. reported that 82 patients are undergoing liver transplantation and 72 patients (88%) developed sarcopenic obesity and metabolic syndrome despite resuming routine exercise after liver transplantation [73]. In LC patients, who receive liver transplantation, appropriate nutrition and exercise after transplantation may be required.

BMI is a simple anthropometric index calculated from individual height and weight and is widely used. However, BMI is limited anthropometrically in that it does not evaluate individual components of body weight such as muscle volume or regional fat distribution. Body mass can be grossly divided into two compartments. These are fat mass and fat-free mass. In a multicompartment body composition model, fat-free mass may be partitioned into skeleton and integument and skeletal muscle and visceral organs and total body water. Total body water is further partitioned into intracellular and extracellular water [74]. Regional fat distribution plays an essential role especially in patients with metabolic syndrome [75]. Taking these into consideration, BMI may not be suitable for evaluating sarcopenic obesity.

## 7. Nutritional Support in LC Patients with Sarcopenia

The aims of nutritional therapy in cirrhotic patients are the support of liver regeneration, the prevention or correction of specific nutritional deficiencies, and the prevention and/or treatment of the LC related complications [76]. The recommendations in nutritional intervention target the optimal supply of adequate substrates related to requirements linked to protein, energy, lipids, carbohydrates, vitamins, and minerals. Early identification and treatment of malnutrition in LC patients have the potential to lead to better clinical outcome and prevent LC related complications [77].

### 7.1. Vitamin

Vitamin deficiencies such as vitamin A, B, D, and E in LC patients are in general associated with disorders of liver function and diminished reserves and with increasing severity of the disease. They are related to inadequate dietary intake and/or malabsorption. Fat soluble vitamin deficiencies are common manifestations in LC patients [78]. Thus, vitamin supplementation may be essential for advanced LC patients [78].

### 7.2. Minerals

Zinc is an essential trace element required for normal cell growth, development, and differentiation and zinc deficiency is common in LC patients [79]. Zinc supplementation is demonstrated to reverse clinical signs of zinc deficiency in LC patients [79, 80]. Furthermore, zinc supplementation produced metabolic effects and trended toward improvements in liver functional reserve, hepatic encephalopathy, and general nutritional status [80, 81].

### 7.3. BCAA

In LC patients, the plasma level of branched-chain amino acid (BCAA) is positively correlated with the serum albumin level. Such a correlation is seen only in patients with chronic liver diseases such as cirrhosis [1, 2, 76]. The albumin-BCAA correlation and the inability of cirrhotic patients to maintain an adequate plasma level of BCAA with diet alone serve as the theoretical rationale for the use of BCAA granules for the treatment of cirrhosis. In cirrhotic patients, BCAA uptake in skeletal muscle is increased for ammonia detoxification and energy production and, in turn, the plasma level of BCAA and albumin production decrease [1, 2, 76]. BCAA granules (LIVACT, Ajinomoto Pharma, Tokyo, Japan) contain L-valine, L-leucine, and L-isoleucine at a ratio of 1.2 : 2 : 1. L-leucine induces albumin synthesis in hepatic cells via transcription factors such as mammalian target of rapamycin (mTOR) [1, 76, 82–88]. BCAA granules were originally developed for the treatment of hypoalbuminemia associated with decompensated cirrhosis. However, later studies found a variety of other pharmacological actions of this drug. BCAA granules therapy not only improves hypoalbuminemia but also inhibits cirrhosis-related complications such as esophageal varices and ascites, reduces insulin resistance and oxidative stress, improves fatty acid metabolism, stimulates the immune system, and inhibits angiogenesis [1, 76, 82–88]. The 2010 guidelines for comprehensive treatment of hepatitis virus-related cirrhosis in Japanese patients recommend the use of BCAA granules to preserve liver function and inhibit hepatic carcinogenesis [89]. Furthermore, Hanai et al. recently reported that BCAA supplementation improved the survival of sarcopenic LC patients in their subgroup analysis (*P* < 0.01) [36]. Conversely, the American Society for Parental and Enteral Nutrition (ASPEN) and the European Society for Clinical Nutrition and Metabolism recommend that BCAA supplementation be carried out only in cirrhotic patients with chronic hepatic encephalopathy that is refractory to pharmacotherapy [90, 91]. There may be differences of indications for BCAA therapy in LC patients between Japan and Western countries.

### 7.4. Carnitine

Carnitine deficiency has been demonstrated to be linked to LC [92]. Administration of L-carnitine, which is a derivative with high bioactivity in carnitine derivatives, has been suggested as a safe alternative treatment for LC patients [93, 94]. In field clinical practice, Malaguarnera et al. reported that LC patients treated with L-carnitine showed greater reductions in serum ammonia levels and improvements of neuropsychological functioning in comparison with placebo [93]. On the other hand, Nakanishi et al. demonstrated that L-carnitine reduces muscle cramps in LC patients [94]. However, whether L-carnitine improves sarcopenia in LC patients remains unclear. Further examination will be needed to confirm these results.

## 8. Sarcopenia, Patient Performance Status, and HCC Prognosis

Severe muscle wasting or sarcopenia is one of the most common and frequently hidden complications in HCC patients with LC, which negatively has an effect on survival and quality of life. These complications may potentially lead to deterioration of performance status (PS). The PS scale measures how the daily living ability is affected by the underlying disease. The PS scale recommended by the Eastern Cooperative Oncology Group (ECOG) is widely used by clinicians to assess the functional status in patients with various cancers [95]. It also serves as an indicator of cancer therapy and predictor of patient survival. The PS scale is a major survival determinant in patients with HCC and is specifically included in the BCLC staging system as an essential parameter for treatment guidance for HCC [96]. In our previous study of PS on survival in HCC patients (*n* = 1003), a worse PS was significantly associated with age, gender, Child-Pugh classification, HCC stage, JIS score, initial treatment option for HCC, maximum tumor size, alanine aminotransferase value, hypoalbuminemia, hyperbilirubinemia, renal insufficiency, hyponatremia, and prothrombin and poorer PS was an independent predictor linked to OS with a hazard ratio of 1.773 (*P* < 0.001). Thus, we concluded that PS was closely associated with status of HCC patients with LC and could be an important predictor for these populations [20]. In our recent another study, we proposed PS combined JIS system in HCC patients with LC and demonstrated that it can be a useful prognostic system for HCC patients complicating with LC as compared with other classification systems such as original JIS system, BCLC, and CLIP (*n* = 1170) [97].

### 8.1. HCC and Impact of Sarcopenia

Fujiwara et al. retrospectively investigated the effect of body composition components on survival in HCC patients (*n* = 1257) and demonstrated that sarcopenia, intramuscular fat (IMF) deposition, and high VSR (called visceral adiposity) were significantly associated with mortality, independent of HCC stage or Child-Pugh classification and their multivariate analysis revealed that sarcopenia (hazard ratio (HR), 1.52; 95% confidence interval (CI), 1.18–1.96; *P* = 0.001), IMF deposition (HR, 1.34; 95% CI, 1.05–1.71; *P* = 0.020), and visceral adiposity (HR, 1.35; 95% CI, 1.09–1.66; *P* = 0.005) but not BMI were significant predictive factors linked to survival [98].

### 8.2. Prognosis in Sarcopenic HCC Patients according to Treatment Modality for HCC

#### 8.2.1. Surgical Resection

The quality of skeletal muscle has attracted much attention as a novel indicator of sarcopenic HCC patients. Recently, Hamaguchi et al. demonstrated in their large study (447 HCC patients) that preoperative quality of skeletal muscle as evaluated by intramuscular adipose tissue content using preoperative CT imaging was well linked to postoperative mortality and HCC recurrence [99].

In addition, a recent study demonstrated that sarcopenia, as assessed by total psoas major volume, was an independent factor predictive of postoperative complications for primary hepatic cancers (HR; 3.06) [100]. Another recent study revealed that, in 109 HCC patients undergoing hepatectomy, sarcopenic HCC patients (*n* = 59) had significantly shorter median overall survival than nonsarcopenic HCC patients (52.3 months* versus* 70.3 months; *P* = 0.015) and, in their multivariate analysis, sarcopenia was revealed to be an independent predictor of poorer overall survival (HR = 3.19; *P* = 0.013) and disease-free survival (HR = 2.60; *P* = 0.001) [101]. Otsuji et al. reported that preoperative sarcopenia increased the morbidity rate including the rate of developing liver failure in patients treated with major hepatectomy with extrahepatic bile duct resection (*n* = 256) [102].

#### 8.2.2. Trancatheter Arterial Therapies

Dodson et al. demonstrated that sarcopenia was an independent predictor of mortality following transcatheter intra-arterial therapy with sarcopenic patients having a twofold increased risk of mortality in patients with liver malignancies (*n* = 216) [103].

#### 8.2.3. Molecular Targeted Therapy

Recently, sarcopenia, regardless of the presence of weight loss, has been identified as an independent adverse predictor for systemic chemotherapy toxicity. Mir et al. reported that in advanced HCC patients with Child-Pugh A (*n* = 40), sarcopenia predicts the occurrence of dose limiting toxicities within the first month of sorafenib therapy [104].

## 9. Conclusion

Several noninvasive methods for evaluating the degree of liver fibrosis and nutritional status have been developed and these methods have been used for predicting prognosis in patients with LC. LC patients often have PEM and poor physical activity. These conditions often result in sarcopenia, affecting negatively the survival. Sarcopenic obesity, which is recently recognized as novel clinical entity, may lead to accelerating sarcopenia progression. Thus, adequate nutritional support and exercise management may be essential for such patients. In HCC patients complicating LC, sarcopenia is also a significant problem due to its prognostic impact (Figure 1).

## Figures and Tables

**Figure 1:**